# Radical Reaction Control in the AdoMet Radical Enzyme CDG Synthase (QueE): Consolidate, Destabilize, Accelerate

**DOI:** 10.1002/chem.201604719

**Published:** 2016-12-13

**Authors:** Christof M. Jäger, Anna K. Croft

**Affiliations:** ^1^ The University of NottinghamDepartment of Chemical and Environmental EngineeringUniversity ParkNottinghamNG7 2RDUnited Kingdom

**Keywords:** catalysis, density functional calculations, radical clocks, radical SAM enzymes, radical stabilisation energies

## Abstract

Controlling radical intermediates and thus catalysing and directing complex radical reactions is a central feature of *S*‐adensosylmethionine (SAM)‐dependent radical enzymes. We report ab initio and DFT calculations highlighting the specific influence of ion complexation, including Mg^2+^, identified as a key catalytic component on radical stability and reaction control in 7‐carboxy‐7‐deazaguanine synthase (QueE). Radical stabilisation energies (RSEs) of key intermediates and radical clock‐like model systems of the enzyme‐catalysed rearrangement of 6‐carboxytetrahydropterin (CPH4), reveals a directing role of Mg^2+^ in destabilising both the substrate‐derived radical and corresponding side reactions, with the effect that the experimentally‐observed rearrangement becomes dominant over possible alternatives. Importantly, this is achieved with minimal disruption of the thermodynamics of the substrate itself, affording a novel mechanism for an enzyme to both maintain binding potential and accelerate the rearrangement step. Other mono and divalent ions were probed with only dicationic species achieving the necessary radical conformation to facilitate the reaction.

## Introduction

Reactive radical intermediates are able to achieve complex chemical conversions that are either inaccessible or extremely difficult to achieve through alternative approaches. Examples include C−C bond^1^ and thioether bond forming reactions,^2^ insertion reactions,^3^ and carbon‐skeleton rearrangements.^4^ One major limitation in carrying out radical reactions is that the benefits incurred by high reactivity are attenuated through lower selectivity of reaction, potentially leading to unwanted by‐products. Nature has overcome this challenge through developing careful mechanisms for the control of radical reactions within enzymes.

Enzyme‐based radical reactions can take many forms, either initiated by metals, oxygen, protein‐based radicals or organo‐metallic species. Both coenzyme‐B_12_‐dependent and radical SAM (or adomet radical) enzymes initiate radical transformations through generating an adenosyl methionine (adomet) carbon‐based radical intermediate **AdoR**, providing a highly‐reactive species to carry out reactions. Systems utilising this intermediate, as generated from coenzyme B_12_, have provided insights into a number of specific mechanisms that can be used to precisely control reactivity both of the intermediate adomet radical and radicals formed during the transformations catalysed by the relevant enzymes. Examples include the concept of electrostatic catalysis^5^ through electrostatic effects between the sugar and the protein and electrostatic stabilisation of leaving groups, selectivity through negative catalysis^6^ preventing undesired side reactions, examples of (retro) push–pull catalysis and barrier lowering effects by partial proton transfer for 1,2‐migration (1,2‐shift) reactions,^7^ and radical‐cage effects.^8^

Radical SAM enzymes provide an even broader spectrum of chemical transformations than the coenzyme B_12_‐dependent enzymes, with the radical SAM superfamily catalysing more than 65 different reactions (corresponding to a search on the structure–function linkage database).^9^ As such, these enzymes have attracted recent, significant interest in their catalytic mechanisms (see for example reviews by Dowling et al.^10^ and Broderick et al.^11^), and have the potential to inform us further on additional chemical mechanisms of radical control that may be employed by nature.

**Scheme 1:**
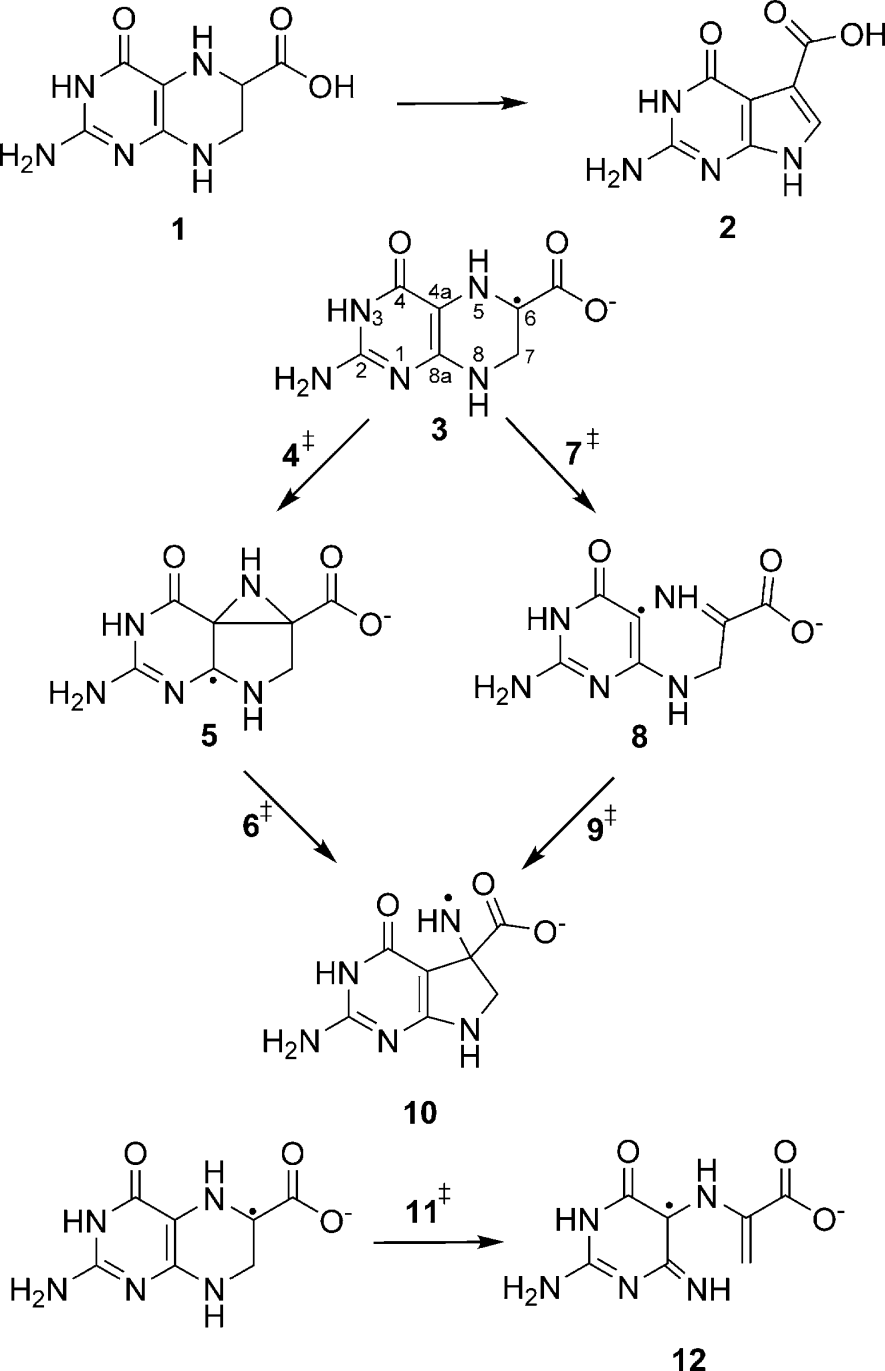
Reaction catalysed in the enzyme QueE and possible rearrangement pathways shown for the anionic radical of CPH4.

One such challenging mechanism features the ring‐contraction reaction catalysed by the enzyme 7‐carboxy‐7‐deazaguanine (CDG) synthase, or QueE (Scheme 1).^12^ This reaction drives a central step of queosine synthesis and facilitates the formation of the 7‐deazapurine scaffold. Deazapurines are found to be widely spread in nature and are of particular interest due to their antibacterial, antifungal, antineoplastic and herbicidal activity.^13^

First structurally described and mechanistically investigated by Bandarian^12^ and Drennan and coworkers,^14^ and very recently also investigated theoretically by means of QM/MM calculations,^15^ the enzyme's catalysis shows a rate determining dependence on Mg^2+^ that is directly involved in the radical rearrangement as a feature never reported for radical SAM enzymes before. The theoretical study by Zhu and Liu,^15^ uses MD snapshot‐based QM/MM calculations to rule out one possible reaction mechanism. They suggest that the predominant effect of the ion is electrostatic, with its role to hold the substrate in its reactive conformation and that the different coordination to Na^+^ might be the main reason for different activities (by making the radical rearrangement step rate determining), leaving the question open as to what is the distinct effect of the metal complexation on the radical intermediate and the reaction as a whole.

We now present a closer look at the radical control of the reaction, namely whether the metal complexation influences the radical reactivity of these highly reactive species and how it is controlled in the enzyme. We have looked at the rearrangement by means of high level ab initio and DFT calculations, focusing on a deeper understanding of the nature of the radicals and how both the metal coordination and the enzyme influence the radical intermediates and guide the catalysis away from unwanted side reactions. We reveal that these factors act in complementary ways by keeping the stability of the radicals and the kinetic barrier of the rearrangement in balance and preventing side reactions that appear to be favoured outside the enzyme active site. We relate our findings to common concepts of enzyme catalysis and highlight once again the observation that there is nothing ‘free’ about ‘free radical intermediates’ in enzyme catalysis.^13^, ^16^

## Results and Discussion

Informed by the experimental findings of Drennan and coworkers^14^ and the very recent QM/MM studies by Zhu and Liu,^15^ two possible radical rearrangement mechanisms for the C6 radical (**3**) of 6‐carboxy‐5,6,7,8‐tetrahydropterin **1** to **10** (Scheme 1) can be envisaged. Isotope‐transfer experiments suggest that the initial hydrogen abstraction from CPH4 occurs from the C6 carbon^14^, ^17^ and that the radical can then either undergo a C4a−C6 bond formation to produce the bridged azacyclopropylcarbinyl intermediate (**5**) followed by C4a−N5 cleavage to deliver the aza radical **10**, or it can initially cleave the C4a−N5 bond, fragmenting to give the ring‐opened imine intermediate **8** with subsequent recombination to the cyclised product **10**.

To obtain a deeper understanding of both the factors influencing the kinetics and the thermodynamics of this radical rearrangement reaction we have now investigated both possible rearrangements with gas phase model calculations at different levels of theory, comparing them to insights from the enzyme directly. Special attention has been paid to the role of cation complexation to the radical intermediate, which plays a crucial role in the enzymatic catalysis,^14^ and the specific nature of the radicals involved in the rearrangement.

In the context of the radical's properties, the thermodynamic stabilisation of the transiently‐formed highly reactive radicals can give insights into how reactive or energized the radicals are and thus how likely and quickly they could undergo unwanted side reactions. The weaker the stabilisation, the more energized the radicals are and thus the more difficult it is to control the specificity of the reaction. In enzymatic radical reactions, one significant role of the enzyme is usually to control the highly reactive intermediates in order to prevent the more favorable side reactions that would appear in enzyme‐free systems. This concept is often referred to as negative catalysis.^6^

The stabilities of the radicals can be calculated using a formal hydrogen abstraction between closed shell precursors (e.g. CPH4) and a reference radical, such as CH_3_^.^ for carbon centered radicals (like **3**) and NH_2_^.^ for nitrogen centered radicals (like **10**), as given in Equations (1), (2).

(1) $$\mathrm{CH}{}_{3}{}^{•}+H-R\to \mathrm{CH}{}_{4}+R{}^{•}$$

(2) $$\mathrm{NH}{}_{2}{}^{•}+H-R\to \mathrm{NH}{}_{3}+R{}^{•}$$

The reaction enthalpy for process (1) is then defined as per Equation (3):

(3) $$\mathrm{RSE}\left(R{}^{•}\right)=H{}_{298}\left(R{}^{•}\right)+H{}_{298}\left(\mathrm{CH}{}_{4}\right)-H{}_{298}\left(R-H\right)-H{}_{298}\left(\mathrm{CH}{}_{3}{}^{•}\right)$$

and is often referred to as the radical stabilisation energy (RSE). Comparing RSEs of the compounds involved in the enzymatic catalysis can provide details of the thermodynamic aspects of the enzymatic catalysis.

The concept of RSE comparison has been used, alongside a list of studies of RSEs and related relative and absolute bond dissociation energies (BDEs), for a diverse set of radicals^18^ including amino acids and peptide model radicals^19^ and other radicals playing a role in enzymatic catalysis, particularly SAM‐mediated catalysis.^20^ These, and further comparative studies,^21^ also present a valuable estimation of the quality of different methods for obtaining reliable radical stabilisation energies. From the computationally more affordable methods, the M06‐2X^22^ and the BMK^23^ hybrid functionals turn out to be best performers for retrieving accurate BDEs and RSEs, when compared with the results of high level calculations such as the G3B3 method.^24^ Thus, these functional approaches deliver a good and computationally less expensive alternative.

In this study, geometry optimisations were carried out at the B3LYP/6‐31+G(d) and the BMK/6‐31+G(2df,p) level of theory, as the latter is also used with the G4(MP2)‐6X method.^25^ Single point calculations at a higher level were additionally carried out on the B3LYP structures and compared to high level G3B3 calculations for the smaller model systems.

Although B3LYP energies have often been shown to underestimate reaction barriers,^26^ previously reported work on the radical reaction in pyruvate formate–lyase, also including a carboxylate moiety, showed exactly the opposite effect.^27^ In the present study an overestimation of energy barriers by B3LYP is observed for both the charged carboxylate systems and for all relative radical stabilisation energies. Overall, the M06‐2X functional showed the best results when compared to G3B3 results, where available. Thus all relative energies discussed in the text correspond to the M06‐2X results and all interatomic distances are given for the BMK results. All relative energies calculated can also be found in the Supporting Information together with the corresponding absolute energies.

Due to the acidity of the carboxylate group of CPH4, this molecule is expected to be deprotonated both in solution and in the protein substrate complex. The crystal structure of QueE (*B. multivorans*) further shows that CPH4 is fixed in position by a salt bridge to an arginine residue (Arg27).^14^ This salt bridge can also provide a partial reprotonation of the bound carboxylate. Smith and coworkers^27^ showed, using the example of pyruvate formate–lyase, how difficult and important it is to assign the most reasonable protonation state in model gas phase calculations of arginine‐bound carboxylates in order to sensibly predict the reaction path and energies. They concluded that the neutral carboxylic acid delivers a better approximation to the salt bridge arrangement than the charged carboxylate. Due to the special nature of the CPH4 substrate, both protonation states have been tested and the results reported here.

### Anionic radical model

**Figure 1:**
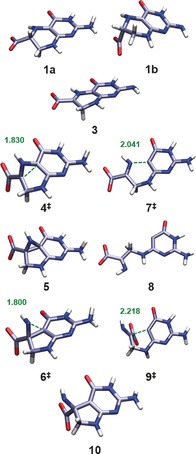
BMK/6‐31+G(2df,p) optimised structures of the deprotonated form of CPH4 (**1 a**, **1 b**) and subsequent radical rearrangement of anionic radical model (**3**). Interatomic distances given in Ångstroms.

The relative energies for the rearrangement of the anionic CPH4 radical are listed in Table 1. We focused on the rearrangement of the *R*‐enantiomer relevant in the biological pathway of Queuosine synthesis. Two low energy conformers (Figure 1) were identified for the anionic CPH4, which differ in the carbon atom pointing out of the plane of the heterocycle. In **1 a** the C7 carbon adopts an *endo* conformation, and **1 b** represents the C6 *endo* conformation, leading to the carboxylate group pointing axial out of the ring plane. The conformer **1 b** is similar to the one found in the crystal structure of QueE^14^ and is slightly less stable by 7.8 kJ mol^−1^ than the corresponding conformer **1 a**. Upon radical formation, the radical center assumes a planar conformation.

**Table 1:** Calculated relative energies for the radical rearrangement of the anionic CPH4 radical.

	BMK/6‐31 +G(2df,p)^[a]^	B3LYP/ 6‐31+G(d)^[a]^	MP2^[b]^	M06‐2X^[b]^
**1 a**	0.0	0.0	0.0	0.0
**1 b**	10.6	11.4	6.8	7.8
**3**	0.0	0.0	0.0	0.0
**4^≠^**	142.0	158.1	160.0	144.6
**5**	115.4	136.9	110.4	110.5
**6^≠^**	134.0	151.5	149.3	139.8
**7^≠^**	195.2	185.4	199.5	183.2
**8**	139.7	127.8	116.6	125.4
**9^≠^**	205.5	193.0	206.1	194.1
**10**	72.5	80.0	55.0	74.4
**11^≠^**	92.9	78.4	61.3	85.6
**12**	35.7	24.7	48.8	29.7

[a] All relative energies are given in kJ mol^−1^; absolute energies are given in the supporting information; B3LYP, MP2, M06‐2X energies are corrected with unscaled B3LYP/6‐31+G(d) zero‐point energies, BMK values with BMK/6‐31+G(2df,p) zero‐point energies respectively. [b] 6‐311++G(3df,3p) basis set.

**Figure 2:**
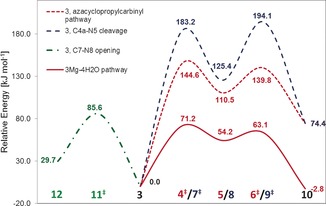
Schematic energy diagram for the rearrangement of the charged CPH4 radical **3** at the M06‐2X level.

The subsequent radical rearrangement affords very high energy barriers for both pathways (see Figure 2 and Table 1). The rearrangement through the azacyclopropylcarbinyl intermediate (**5**) has a C−C bond formation barrier of 144.6 kJ mol^−1^ in the transition state **4^≠^** and a subsequent ring opening barrier of 29.3 kJ mol^−1^ in the corresponding transition state **6^≠^**. The ring opening/closing reaction through this latter pathway is not competitive with even higher barriers of 183.2 (**7^≠^**) and 68.7 kJ mol^−1^ (**9^≠^**). Moreover, the overall reaction energy appears to be highly endothermic by 74.4 kJ mol^−1^. On the other hand, the ring opening through homolytic cleavage of the N8−C7 bond to form the amino acrylate‐based radical species (**12**) only has a transition barrier of 85.6 kJ mol^−1^ (**11^≠^**) and also a much lower endothermicity. Thus, it appears that this ring opening—which is not followed by a subsequent rearrangement—is kinetically and thermodynamically favoured in gas phase.

### Neutral radical model

For the radical rearrangement of the neutral model, several different orientations of the carboxylate hydrogen atom are possible. We have investigated the two possible *syn*‐planar carboxylic acid conformations, **3 H_cis_** and **3 H_trans_**_,_ with the carboxylic hydroxyl in a *cis* or *trans* conformation, respectively, relative to N5 (see also Scheme S1 in the Supporting Information). We have neglected the *anti*‐planar conformations since they would be less favourable for building the crystallographically identified salt bridge to the arginine in the active site of QueE. The relative energies of the rearrangements are listed in Table 2.

**Table 2:** Calculated relative energies for the radical rearrangements of the neutral substrate **3 H** and model **13 H**.

	BMK/6‐31 +G(2df,p)^[a]^	B3LYP/ 6‐31+G(d)^[a]^	MP2^[b]^	M06‐2X^[b]^	G3B3
**3 H_cis_**	0.0	0.0	0.0	0.0	
**4 H^≠^**_**cis**_	177.5	178.0	173.0	162.7	
**5 H_cis_**	176.4	158.9	166.6	156.2	
**6 H^≠^**_**cis**_	185.0	169.2	178.7	173.5	
**10 H_cis_**	119.0	109.1	96.3	111.8	
**3 H_trans_**	0.5	0.5	−2.6	0.2	0.0
**4 H^≠^**_**trans**_	162.6	180.4	173.5	164.8	159.2
**5 H_trans_**	162.6	179.3	184.2	158.3	151.3
**6 H^≠^**_**trans**_	170.9	187.2	181.1	175.3	150.9
**7 H^≠^**_**trans**_	271.5	250.5	298.2	259.7	151.3^[c]^
**10 H_trans_**	112.4	121.7	96.7	113.7	92.5
**13 H_trans_**	0.0	0.0	0.0	0.0	0.0
**14 H^≠^**_**trans**_	145.0	145.8	110.8	128.7	123.1
**15 H_trans_**	115.5	140.3	65.9	105.9	104.8
**16 H^≠^**_**trans**_	119.3	143.3	67.9	107.9	106.9
**17 H_trans_**	107.0	117.7	77.1.	102.6	111.4

[a] All relative energies are given in kJ mol^−1^; absolute energies are given in the supporting information; B3LYP, ROMP2, M06‐2X energies are corrected with unscaled B3LYP/6‐31+G(d) zero‐point energies, BMK values with BMK/6‐31+G(2df,p) zero‐point energies respectively. [b] 6‐311++G(3df,3p) basis set. [C] nimag=0.

The *cis* and *trans* isomers are almost identical in energy and both show higher and very similar rearrangement barriers in comparison to the anionic model. The barriers for the azacyclopropylcarbinyl ring opening are 162.7 (**4 H^≠^**_**cis**_) and 164.8 kJ mol^−1^ (**4 H^≠^**_**trans**_), respectively, with a further increase in the endothermic character of the rearrangement. The ring opening mechanism is not competitive, with a barrier of 259.7 kJ mol^−1^ for **7 H^≠^**_**trans**_. Structurally, the neutral radicals do not show significant differences when compared with the anionic radicals, except from an increase in the bond breaking and forming distances in the transition states (see Supporting Information Table S4 for details).

**Scheme 2:**
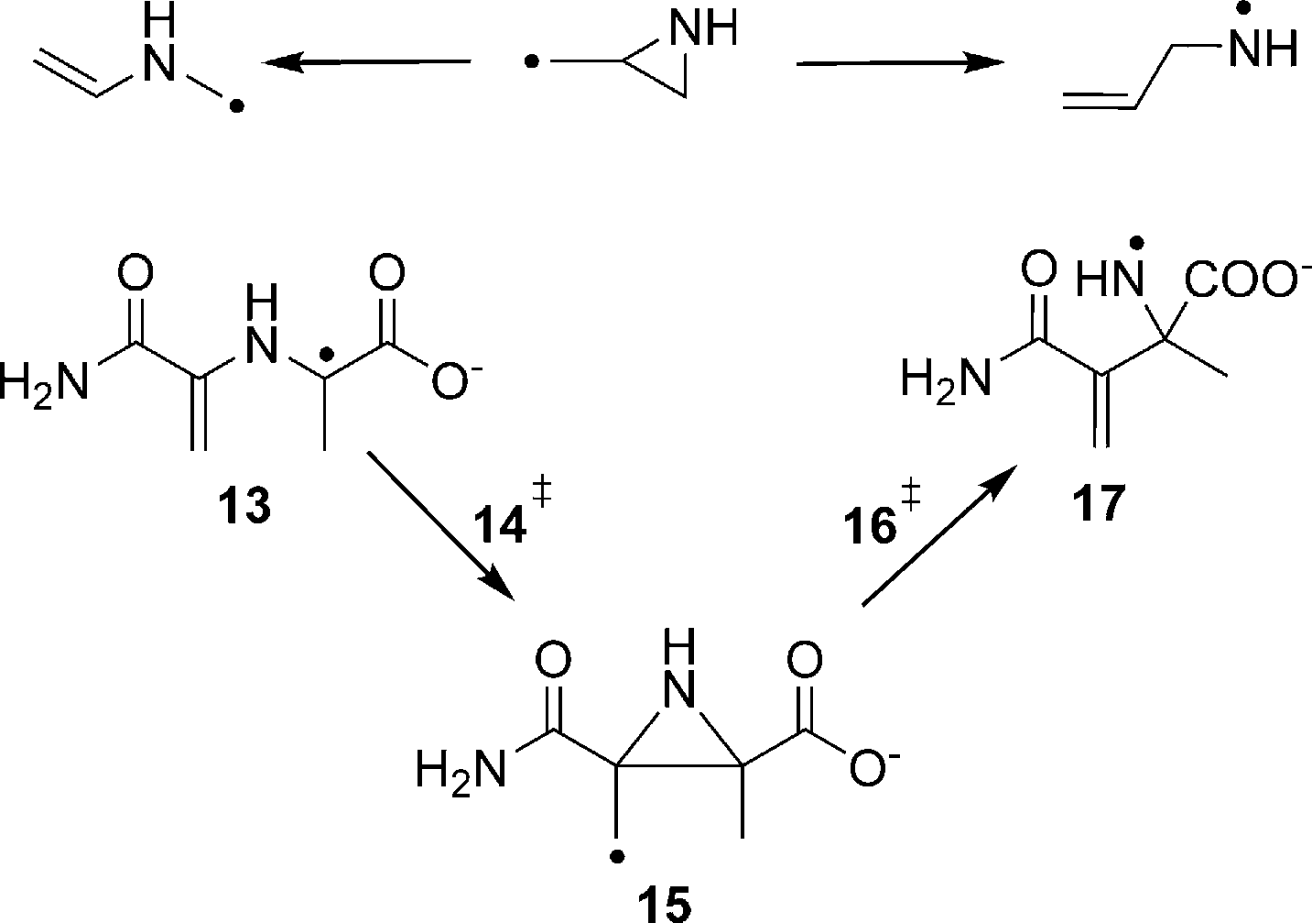
2‐Aziridinylcarbinyl ring opening reaction (top). Rearrangement of model **13** (bottom).

A closer examination of the intermediate (**3**, **3 H**) shows that it incorporates a substituted 2‐aziridinylcarbinyl radical (Scheme 2, top) motif, which equates to a hetero‐substituted cyclopropylcarbinyl radical. The ring opening reactions of such radicals are one of the fastest class of unimolecular reactions known.^28^ This group of extremely fast radical rearrangements have also been used extensively as radical clocks,^29^ since they can be used as diagnostic probes for radical reactions of unknown rate in chemical and biochemical systems.^30^ As such, they have been targets of various theoretical studies^31^ including investigations on the effect of complexed ions on the clock's kinetics.^32^

**Figure 3:**
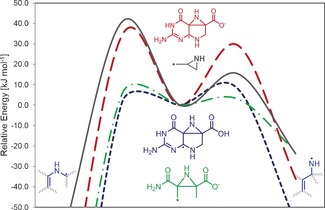
Schematic energy diagram depicting the substituent effect for the radical rearrangement barrier of the aziridinylcarbinyl radical clock. Data for the allylaza radical clock adopted for comparison from Smith et al.31i

High‐level theoretical calculations by Smith et al.31i on the unsubstituted aziridinylcarbinyl radical clock have shown that the possible ring opening to the allylaza radical (Δ*H* ^≠^(CBS‐RAD)=15.4 kJ mol^−1^), corresponding to the CPH4 radical **3**, and the opening to the vinylazamethyl radical (Δ*H* ^≠^(CBS‐RAD)=40.8 kJ mol^−1^), corresponding to **10**, differ significantly in their rearrangement barriers (see Figure 3). Our results found that the rearrangement of **3** shows the same trend with barriers of 29.3 and 34.1 kJ mol^−1^, respectively, with an increased barrier for the allylaza radical formation. However, looking at the neutral model this trend is reversed with barriers of 17.0 (via **6 H^≠^**_**trans**_) and 6.5 kJ mol^−1^ (via **4 H^≠^**_**trans**_) (see Table 2). Thus, the barrier for the vinylazamethyl radical formation almost vanishes. Although the substitution and structural constraints of the CPH4 system seem not to significantly influence the kinetic rearrangement behavior of the charged system, it does reverse the kinetics for the neutral system, illustrating how significant substitution effects can be. Also the relative stabilities of the endpoints to each other and also to the radical clock intermediate differ significantly. The calculated energy differences between intermediate **5** and radicals **3** and **10**, respectively, are almost twice as high as those calculated for the corresponding radicals in the radical clock investigated by Smith et al. (38.1 kJ mol^−1^)31i which highlights once again the strong effect of substituents on the stability of the radicals.

### Radical stabilisation

The thermodynamic difference for the rearrangement of the differently substituted aziridinylcarbinyl radical clocks is highlighted by the stabilisation of the CPH4 radical (see Table 3). The neutral radical **3 H_cis_** has an extremely high RSE of −139.6 kJ mol^−1^ (−133.2 kJ mol^−1^for **3 H_trans_**), whereas the carboxylate **3** is comparatively less stable with an RSE of −86.2 kJ mol^−1^, although still very stable. The origin of this high stability lies in several contributions. One major contributor arises from captodative stabilisation19b, 33 of the radical center by the π‐electron‐donating (HNR) and π‐electron‐accepting (COOH) substituents. This captodative effect is significantly lower when the carboxylic acid is deprotonated. The other stabilising influence is based on the delocalisation of the unpaired electron within the ring‐system of CPH4. The substrate radicals show a relatively high delocalisation, indicated by relatively low Mulliken unpaired spin densities on the radical centers of 0.65 for **3** and 0.48 for **3 H**, respectively (see Supporting Information Table S5 for details). The spin delocalisation increases in the intermediate, before localising almost completely in the N‐centered product radical **10**.

**Table 3:** Calculated radical stabilisation enthalpies at 298.15 K.

	BMK/ 6‐31+G(2df,p)^[a]^	B3LYP/ 6‐31+G(d)^[a]^	MP2^[b]^	M06‐2X^[b]^	G3B3
**3**	−95.6	−104.9	−49.0	−86.2	−86.9
**3 H_cis_**	−148.7	−157.6	−117.2	−139.6	
**3 H_trans_**	−141.3	−152.1	−113.0	−133.2	‐−120.7
**10**	−51.2	−53.2	−24.3	−43.1	
**10 H_cis_**	−36.4	−37.4	−22.4	−30.3	
**10 H_trans_**	−29.0	−30.5	−16.8	−23.6	−20.9
**8**	46.4	25.7	70.3	41.9	
**13**	−92.2	−99.7	−42.0	−81.1	−73.2
**13 H_trans_**	−116.0	−126.9	−68.4	−110.6	−100.8
**17**	−35.3	−44.1	6.8	−24.4	
**17 H_trans_**	−22.4	−33.3	−6.6	−26.4	−20.8

[a] All radical energies are given in kJ mol^−1^; absolute energies are given in the supporting information; B3LYP, ROMP2, M06‐2X energies are corrected with B3LYP/6‐31+G(d) enthalpy corrections (BMK values with BMK/6‐31+G(2df,p) enthalpy corrections respectively). [b] 6‐311++G(3df,3p) basis set.

The energy barriers and RSEs for the model radical **13** (see Scheme 2) have been calculated to identify possible ring strain effects and explore the role of spin delocalisation. The rearrangement barrier of 128.7 kJ mol^−1^ for the intermediate **14 H^≠^**_**trans**_ is roughly 36 kJ mol^−1^ lower in comparison to its CPH4 counterpart, with the radical stabilisation reduced by 20 kJ mol^−1^. The spin density is also delocalised in the substrate radical. However, although the delocalisation for the CPH4 radical **3** was spread in the ring system, including N5 for the neutral species, it is shared predominantly between C6 and C8a for the radicals **13**/**13 H**. In contrast to the CPH4 rearrangement, the unpaired spin density is highly localised on the C8a atom for the intermediate **15**/**15 H**. Still, the rearrangement appears to be slightly more favorable for the model system.

In comparison to other radical SAM enzymes, the obtained RSE values for the substrate radical appear to be extremely high even in comparison to C_α_‐amino acid radicals generated by the important rSAM family subclass of glycyl radical activating enzymes (GRAE),^34^ which can achieve a very similar captodative stabilisation.

Theoretical studies on C_α_‐amino acid radicals19e,19f, 35 and peptide models19a,19b have revealed that a thermodynamic reason for the choice of glycyl peptide radicals lies in their more stable nature in comparison to other amino acid radicals.19a This higher stability prevents fast side reactions in which hydrogen atoms might be otherwise abstracted from adjacent residues. Similarly, the higher stability of glycyl radicals with respect to other amino acid radicals, is balanced by additional factors. Even though stabilisation of the radical by electron‐donating substitutents at the C_α_ position is achieved, the higher conformational freedom and thus larger accessible conformational space for glycyl radicals free of steric interactions seems to mitigate this effect.19a

### Counterion‐complexed radical models

The energetics of the radical rearrangement change significantly once the substrate is complexed by a counterion. Table 4 shows the results for the complexation of CPH4 radical to a magnesium ion and water molecules. There is a lowering of the activation barrier, relative to the uncomplexed species, by more than one half, to 71.2 kJ mol^−1^ for the model with four water molecules (**3 Mg_4H2O_**), 67.9 kJ mol^−1^ in complex with three waters, and 80.8 kJ mol^−1^ in the charge‐neutral model with two bound waters and one hydroxyl group. Thus, the barrier‐lowering effect is not significantly influenced by the complexation geometry of the magnesium ion and the calculations seem not to be influenced by the charge of the system. The intermediate (**5 Mg**) is much more difficult to locate, as its potential energy well is much shallower. The thermodynamics of the rearrangement, once complexed, change from a strongly endothermic reaction to a slightly exothermic/endothermic reaction with reaction energies between −2.8 and +7.3 kJ mol^−1^ for the different models.

**Table 4:** Calculated relative energies for the radical rearrangements complexed with Mg^2+^.

	BMK/ 6‐31+G(2df,p)^[a]^	B3LYP/ 6‐31+G(d)^[a]^	MP2^[b]^	M06‐2X^b]^
**3 Mg_4H2O_**	0.0	0.0	0.0	0.0
**4 Mg_4H2O_** ^**≠**^	54.7	76.8	76.4	71.2
**5 Mg_4H2O_**	46.7	64.5	55.8	54.2
**6 Mg_4H2O_** ^**≠**^	48.7	67.3	69.5	63.1
**10 Mg_4H2O_**	−14.5	−1.3	−9.6	−2.8
**3 Mg_3H2O_**	0.0	0.0	0.0	0.0
**4 Mg_3H2O_** ^**≠**^	54.3	74.0	75.5	67.9
**5 Mg_3H2O_**	41.8	60.7	51.5	48.9
**6 Mg_3H2O_** ^**≠**^	45.3	64.5	67.2	59.5
**10 Mg_3H2O_**	−14.3	−0.6	−10.2	−2.7
**11 Mg_3H2O_** ^**≠**^	136.7	110.4	170.8	137.6
**12 Mg_3H2O_**	72.0	51.9	124.0	70.2
**3 Mg_2H2O−OH_**	0.0	0.0	0.0	0.0
**4 Mg_2H2O−OH_** ^**≠**^	60.2	81.8	86.0	80.8
**5 Mg_2H2O−OH_**	41.2	63.1	66.6	55.7
**6 Mg_2H2O−OH_** ^**≠**^	48.6	69.4	63.0	71.1
**10 Mg_2H2O−OH_**	−11.9	3.5	−5.3	7.3

[a] All relative energies are given in kJ mol^−1^; absolute energies are given in the supporting information; B3LYP, MP2, M06‐2X energies are corrected with unscaled B3LYP/6‐31+G(d) zero‐point energies, BMK values with BMK/6‐31+G(2df,p) zero‐point energies respectively. [b] 6‐311++G(3df,3p) basis set.

**Figure 4:**
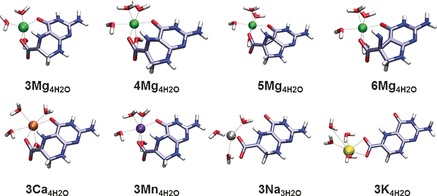
BMK/6‐31+G(2df,p) optimised structures of the rearrangement of the anionic CPH4 radical complexed by Mg^2+^ (top) and CPH4 radical complexed to other cations (bottom).

Similar to the findings in the crystal structure^14^ the magnesium ion is complexed to the C4 carbonyl oxygen and one carboxylate oxygen (see Figure 4), which brings the substrate radical into the conformation represented by structure **1 b** (note that without additional water molecules the complexation also includes the N5 nitrogen). However, although the energy difference for the two substrate conformations **1 a** and **1 b** is minor, there is a very significant effect on the stability of the radical.

The RSE values for the complexes listed in Table 5 show that, along with the reaction barrier, the radical stabilisation energies of the substrates decrease significantly by roughly two thirds. The RSEs of the substrate radicals (**3 Mg**) have been reduced to values between −28.6 and −35.3 kJ mol^−1^ and are now much closer to those of the product radicals (**10 Mg**), with values between −24.0 and −37.9 kJ mol^−1^. The product radicals (**10 Mg**) also experience a slight destabilisation upon complexation.

**Table 5:** Calculated radical stabilisation enthalpies at 298.15 K.

	BMK/ 6‐31+G(2df,p)^[a]^	B3LYP/ 6‐31+G(d)^[a]^	MP2^[b]^	M06‐2X^[b]^
**3 Mg_4H2O_**	−33.0	−46.9	−16.3	−28.6
**10 Mg_4H2O_**	−32.4	−34.2	−20.6	−27.3
**3 Mg_3H2O_**	−37.0	−52.0	−17.3	−33.4
**10 Mg_3H2O_**	−30.0	−30.5	−17.7	−24.0
**12 Mg_3H2O_**	36.8	2.4	109.2	39.3
**3 Mg_2H2O−OH_**	−40.3	−53.5	−17.0	−35.3
**10 Mg_2H2O−OH_**	−36.8	−38.0	−23.9	−37.9
**13 Mg_4H2O_**	−68.8	−80.2	−30.8	−64.8
**17 Mg_4H2O_**	−26.8	−25.0	−30.0	−25.8
**10**	72.5	80.0	55.0	74.4
**11^≠^**	92.9	78.4	61.3	85.6
**12**	35.7	24.7	48.8	29.7

[a] All radical energies are given in kJ mol^−1^; absolute energies are given in the supporting information; B3LYP, ROMP2, M06‐2X energies are corrected with B3LYP/6‐31+G(d) enthalpy corrections, BMK values with BMK/6‐31+G(2df,p) enthalpy corrections respectively.[b] 6‐311++G(3df,3p) basis set.

The reason for the reduction in radical stability lies in the distortion of the planarity of the radical center, which in turn lessens the captodative stabilisation, by poorer alignment of the orbitals required for delocalisation of the unpaired electron density. This less pronounced spin delocalisation is reflected by the higher spin densities observed for the C6 carbon atom of around 0.78 for **3 Mg_XH2O_** and 0.82 for **3 Mg_2H2O−OH_**, respectively (see Supporting Information for details).

As a consequence of the magnesium binding, the substrate is brought into a conformation leading to a much less stable radical substrate closer to the conformation of the transition state, in turn leading to a significant barrier lowering effect. This is consistent with the classical view of catalysis by transition state stabilisation. Such an increase in reactivity, which comes with the decrease in stability, is less expected for a selective radical reaction, in which side reactions need to be minimised. The driving force in this case seems to come from the need to break the planarity of the radical centre in order to proceed through the ring contraction, which comes with a very high energy barrier.

Alternatively, the ring can be opened between atom C7 and N8. The energy barrier of this ring opening increases in complex with Mg^2+^ by roughly 42 kJ mol^−1^ to 137.7 kJ mol^−1^(**11 Mg_3H2O_** ^**≠**^), making this reaction kinetically less favourable. This means that the unwanted side reaction, which is favoured in gas phase, is also prevented by magnesium complexation.

The reaction of the model system affords an energetic landscape similar to, but not as pronounced as that for the CPH4 rearrangement (see Table 6). The rearrangement barrier drops to 69.4 kJ mol^−1^ (**14 Mg_4H2O_** ^**≠**^) on complexation and now is aligned with the corresponding rearrangement barrier for the CPH4 radical. However, the reaction is still very endothermic (51.7 kJ mol^−1^). This change in comparison to the CPH4 system can also be extracted from the RSE values (Table 5), which indicate increased stability for the complexed radical **13 Mg_4H2O_** and lower stability for radical **17 Mg_4H2O_**, compared with the radicals **3 Mg_4H2O_** and **10 Mg_4H2O_**, respectively.

**Table 6:** Calculated relative energies for the radical rearrangements of **14** complexed with Mg^2+^.

	BMK/ 6‐31+G(2df,p)^[a]^	B3LYP/ 6‐31+G(d)^[a]^	MP2^[b]^	M06‐2X^[b]^
**13 Mg_4H2O_**	0.0	0.0	0.0	0.0
**14 Mg_4H2O_** ^**≠**^	62.1	83.5	53.4	69.4
**15 Mg_4H2O_**	53.9	78.5	18.6	50.2
**16 Mg_4H2O_** ^**≠**^	96.6	109.2	97.0	100.3
**17 Mg_4H2O_**	18.8	31.7	22.7	51.7

[a] All relative energies are given in kJ mol^−1^; absolute energies are given in the supporting information; B3LYP, ROMP2, M06‐2X energies are corrected with unscaled B3LYP/6‐31+G(d) zero‐point energies (BMK values with BMK/6‐31+G(2df,p) zero‐point energies respectively).[b] basis set 6‐311++G(3df,3p).

### Effects of other ions

**Figure 5:**
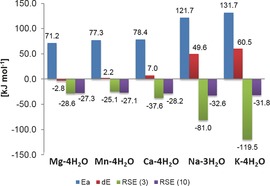
Rearrangement barriers (Ea), reaction energies (dE) and RSE values for **3** and **10** in complex with different ions. Energies at the M06‐2X level are corrected with unscaled B3LYP/6‐31+G(d) zero‐point energies. RSE values are corrected with the corresponding enthalpy correction at 298.15 K.

The model calculations already reveal a catalysing effect for the ring contraction effected by Mg^2+^ complexation, without needing further support by the protein active site. However, from the enzyme studies, an increased reaction rate was found in the presence of Mg^2+^ ions, but not in the presence of other ions.^17^ The rearrangement in complex with other ions was calculated and the resulting energy barriers and net reaction energies can be found in Figure 5.

Out of the set of chosen ions, the rearrangement barrier is lowest for magnesium complexation. The two divalent ions, manganese and calcium show a slight barrier increase, whereas the two monovalent alkaline ions Na^+^ and K^+^ show a high barrier. A closer look at the complexation geometry (see Figure 4) reveals that the divalent ions are able to complex both the carbonyl and the carboxylate oxygen, bringing the radical into the bent conformation. The monovalent ions are only able to complex both oxygens in the product radical, but not in the substrate. Thus, the radical can stay in its preferred planar conformation, which leads to a much better radical stabilisation, at the cost of a high rearrangement barrier. Note also, that the model system with Na^+^ only has three water molecules bound. A similar model containing four water molecules shows an even higher rearrangement barrier, but pushes one of the water molecules into the second solvation shell. The results for this system can be found in the Supporting Information.

The low rearrangement barrier for the magnesium‐complexed CPH4 radical is also in agreement with the recent QM/MM study by Zhu and Lu on this ring contraction in QueE^15^ in which they concluded that the radical rearrangement is not rate limiting when CPH4 is complexed to Mg^2+^ in the active site, but it becomes rate limiting when Na^+^ is present in the active site. Their observed barrier lowering 57 kJ mol^−1^ is similar in magnitude to our findings, opening up the question of the protein's contribution to this step of the reaction.

The experimental findings suggest an increased activity between 3‐fold (QueE from *B. multivorans*) and 10‐fold (QueE from *B. subtilis*) of the enzyme in presence of an excess of Mg^2+^.^14^, ^17^ Further, the crystal structures of QueE from *B. multivorans* also indicate that the coordination environment of the counter‐ion and the hydrogen bonding network of the water molecules and the protein differs for different ions, particularly highlighting the ability of Mg^2+^ to coordinate the carbonyl and the carboxyl moiety of CPH4.^14^ Importantly, it should also been noted that no ions were found in the active site when the substrate analogue 6‐carboxypterin is bound. Thus, the authors concluded, that substrate binding generates the metal binding site.^14^, ^17^ This finding in principle indicates that the metal binding site is not a permanent metal binding site, which in turn suggests that the ions are likely to already be in complex with the substrate upon binding.

Our findings suggest that the catalysis of the rearrangement would also be slower with calcium or manganese ions (neglecting possible effects of the ions on other reaction steps), however not to the extent to which the experimental results indicate. Together with the observation of different magnitudes of increased activity with Mg^2+^ in different QueE enzymes, it could be a matter of how well a specific QueE enzyme can incorporate the substrate in complex with other ions like Ca^2+^. This most importantly includes the ability of the enzyme to position the substrate adequately for the initial hydrogen abstraction. The role of dynamics in the catalysis is until now not clarified, especially how it may be different for different ions.

### Effect of ion complexation on thermodynamic rearrangement profile

As highlighted above, the gas phase model cannot adequately reflect the kinetics for the rearrangement of CPH4 to CDG. The reaction pathway is strongly affected by magnesium complexation and comparing our results to QM/MM studies^15^ suggests that this effect is not further enhanced by the protein itself. On the other hand both the gas phase and the ion complexed model cannot inform about the energy barriers of hydrogen abstraction and re‐abstraction steps. Those are dictated by the enzyme's active site and especially through the ability of the protein to position the substrate in the perfect manner for this selective attack.

**Scheme 3:**
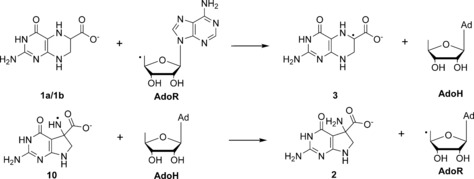
Model for H‐abstraction from deprotonated CPH4 (**1 a/1 b**) and re‐abstraction from product radical **10**.

The model calculations can still be used to assess the thermodynamics of the radical rearrangement and look at the influence of metal binding. This can give us insights into the mechanistic limits for the reaction or, in other words, the feasibility of the rearrangement in general, whether it needs to overcome an energetically demanding path, and whether this could be the rate limiting factor of the whole catalysis. This kind of thermodynamic analysis has been carried out for a set of reactions of different SAM enzymes by Hioe and Zipse.^20^ using the deoxyadenosyl radical (dAdoR) deoxyadensolyl (dAdoH) system as a model reference (see Scheme 3). In this way the reaction enthalpies can be calculated through calculating the corresponding RSE values and combining them with experimental measured bond dissociation energies for H−CH_3_ (439.3 kJ mol^−1^) and H−NH_2_ (450.1 kJ mol^−1^).^36^

For the initial hydrogen abstraction of the gas phase reaction, an RSE‐based analysis yields a strongly exothermic reaction with Δ*H*_298_=−86.2 kJ mol^−1^ for **3** and even higher for **3 H_trans_**. The re‐abstraction of the hydrogen from **10**, in contrast, has an endothermic reaction enthalpy of 25.4 kJ mol^−1^. In comparison to Zipse's findings, the exothermic character of the initial H‐abstraction is much higher than in any other previously examined case.

**Figure 6:**
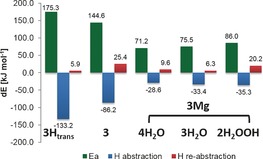
Activation energies (Ea) and thermochemical energy changes of both H‐abstraction steps at the M06‐2X level using dAdoH/dAdoR as reference.

The impact of metal complexation on the radical stabilisation energies is emphasised by examination of the thermodynamic reaction profile. As can be seen in Figure 6, the reaction profile changes upon ion complexation. The exothermic character of the first H‐abstraction is reduced to a value of −28.6 kJ mol^−1^ (**3 Mg_4H2O_**) and the endothermic character of the second H‐abstraction is reduced to 9.6 kJ mol^−1^ (**10 Mg_4H2O_**). Thus, the metal complexation tunes the thermodynamic character of the reaction by preventing the substrate radical from falling into a very stable energetic well, with no easy way to escape. This trap is avoided by the enzyme by combining reaction catalysis with a conformational distortion of the radical intermediate in a way that provides a substantial enough destabilisation to allow the reaction to proceed. In effect it makes the energy profile smoother and easier to overcome.

## Conclusion

The radical ring contraction step catalysed by the enzyme QueE provides a perfect example of the different approaches that may be utilised to control the reactivity of radical species in order to perform a single distinct reaction and to prevent unwanted side reactions.

Ab initio and DFT calculations on gas‐phase model systems to assess the kinetic barriers of the radical rearrangement with calculations of radical stabilisation energies offer an attractive way to assess the stability of highly reactive radicals and the thermodynamics of their reactions. The results reveal that the major contributor to catalysing the rearrangement lies in the complexation of a magnesium ion to CPH4. Consistent with the conclusions of Zhu and Liu's QMMM study,^15^ the magnesium effect is mainly electrostatic and brings the substrate into the preferred reactive conformation. Our results reveal that this reactive conformation shows only a minor energy difference for the substrate, but a very severe one for the radical and that it is this strong effect on the radical that is controlling, directing and accelerating the radical reaction during catalysis.

The Mg^2+^ complexation not only brings the substrate into the reactive conformation but also, once a hydrogen atom is abstracted from the substrate, holds the resultant intermediate radical in a highly strained conformation.

The distortion of planarity at the radical center reduces the radical delocalisation, decreasing the stability of the radical significantly. The bent conformation also mimics the structure of the transition state more closely, illustrative of classical catalysis by lowering the relative energy of transition state. This finding is slightly surprising, since controlling free radicals in enzymes is often attributed to negative catalysis,^6^ through stabilisation of the desired intermediate radical in order to prevent further stabilising side reactions. Indeed, the ring opening side reaction to form the ring‐opened radical **12** is prevented. This reaction would normally be energetically favoured in the gas phase, and is blocked by complexation of the magnesium ion.

Other divalent ions investigated are not hugely inferior to Mg^2+^ in its ability to complex the substrate and thus lower the rearrangement barrier. The alkaline ions Na^+^ and K^+^, however, are not able to stabilise the strained substrate radical conformation required for catalysis. Together with experimental findings showing variation in the magnitude by which an excess of Mg^2+^ ion speeds up the kinetics of different QueE enzymes, our results imply that slight differences in the ion binding sites of the different QueEs could account for their differing ability to facilitate the catalysis for CPH4 when complexed with other ions.

Finally, this study also illustrates the effectiveness of radical stabilisation energies for the evaluation of the thermodynamics of radical reactions in enzymes, provided that the roles of any cofactors and special structural features are already understood. If the catalysis of the radical rearrangement includes a non‐naturally occurring radical conformation—like in this case—assessing correct thermodynamics is not possible by single molecule calculations without additional structural data. Thus, for such systems model systems need to be chosen carefully, for which detailed kinetic and/or structural information from biological studies needs to be present.

## Computational Methods

All DFT calculations reported were carried out with the Gaussian suite of programs.^37^ All geometry optimisations and frequency calculations of the open‐shell systems were performed at the UB3LYP^38^/6‐31+G(d) and the UBMK^23^/6‐31+G(2df,p) levels, including diffuse functions,^39^ and on their restricted counterparts for the closed‐shell systems. Minima and transition states were confirmed by calculating their normal vibrations at the corresponding level of theory. Higher level single point calculations were performed on all B3LYP geometries at the MP2^40^/6‐311++G (3df,3p) and M06‐2X^22^/6‐311++G(3df,3p) level. All relative energies were corrected with unscaled zero‐point energies on the level of their geometry optimisation. The results were compared for the smaller model systems with calculations at the more accurate G3B3^24^ approach. Radical stabilisation energies were calculated according to the procedure outlined above. The energies are calculated applying thermal corrections to enthalpies at 298.15 K at the level of their geometry optimisation. The choice of this enthalpic correction supports an easier comparison with previously reported RSE data. All absolute energies are also reported in the supporting information, together with relevant interatomic distances and spin densities.

## Supporting information

As a service to our authors and readers, this journal provides supporting information supplied by the authors. Such materials are peer reviewed and may be re‐organized for online delivery, but are not copy‐edited or typeset. Technical support issues arising from supporting information (other than missing files) should be addressed to the authors.

Supplementary
