# Supplementary material for: Radical Reaction Control in the AdoMet Radical Enzyme CDG Synthase (QueE): Consolidate, Destabilize, Accelerate
Source: Chemistry. 2016 Dec 13;23(4):953–62. doi: 10.1002/chem.201604719 (PMC5347944; doi:10.1002/chem.201604719)
Supplement: Supplementary file 1 — Supplementary [file CHEM-23-953-s001.pdf]

CHEMISTRY
A European Journal

Supporting Information

Radical Reaction Control in the AdoMet Radical Enzyme CDG Synthase (QueE): Consolidate, Destabilize, Accelerate

Christof M. Jäger* and Anna K. Croft*[a]

chem_201604719_sm_miscellaneous_information.pdf

Supporting Information

Table S1. Detailed results of *ab initio* and DFT calculations. B3LYP/6-31+G(d) optimizations.

Table S2. Detailed results of *ab initio* and DFT calculations. BMK/6-31+G(2df,p) optimizations.

Table S3. Detailed results of *ab initio* and DFT energy calculations.

Table S4. Important interatomic distances. Units in Ångstrom.

Table S5. Mulliken atomic Spin densities >0.1.

Table S6. Detailed results of *ab initio* and DFT calculations. G3B3 results.

Table S7. Calculated relative energies for the radical rearrangements of **3** complexed to different ions.

Table S8. Calculated radical stabilization enthalpies for the rearrangement of **3** complexed to different ions at 298.15 K.

Scheme S1. Conformer nomenclature of CPH4.

Additionally, QM calculation output files and molecular structures can be found on figshare:

Jaeger, Christof (2016): Supplementary material for "Radical Reaction Control in the AdoMet Radical Enzyme CDG Synthase (QueE): Consolidate, Destabilize, Accelerate". figshare.

https://dx.doi.org/10.6084/m9.figshare.c.3574113.v1

Table S1. Detailed results of *ab initio* and DFT calculations. B3LYP/6-31+G(d) optimizations.

B3LYP/6-31+G(d)							
Name	Electronic energy [Hartree]	ZPE [Hartree]	cH [Hartree]	cG [Hartree]	nimag	dE Etot+ZPE [KJ/mol]	RSE Etot+cH [KJ/mol]
**1a**	-771.1292190	0.1704312	0.183987	0.131174	0	0.0	
**1b**	-771.1253198	0.1708649	0.184459	0.131174	0	11.4	
**3**	-770.4927925	0.1568494	0.170626	0.116831	0	0.0	-104.9
**4**	-770.4307542	0.1550377	0.168316	0.115511	1	158.1	
**5**	-770.4394744	0.1556640	0.169394	0.115659	0	136.9	
**6**	-770.4329819	0.1547460	0.168099	0.115314	1	151.5	
**7**	-770.4194935	0.1541816	0.167977	0.113532	1	185.4	
**8**	-770.4424673	0.1551995	0.170017	0.111897	0	127.8	25.7
**9**	-770.4166968	0.1542722	0.168131	0.113853	1	193.0	
**10**	-770.4601402	0.1546542	0.169002	0.113256	0	80.0	-53.2
**(10-H)**	-771.1173907	0.1698691	0.183953	0.129815	0	29.6	
**11**	-770.4602438	0.1541598	0.114271	0.114271	1	78.4	
**12**	-770.4810930	0.1545484	0.168888	0.111980	0	24.7	
**1aHcis**	-771.6605858	0.1835654	0.197543	0.143416	0	4.8	
**3Hcis**	-771.0449564	0.1709745	0.184884	0.131095	0	0.0	-157.6
**10Hcis**	-770.9969400	0.1685024	0.182797	0.127890	0	119.6	-37.4
**(10H-H)cis**	-771.6597591	0.1829809	0.197296	0.142945	0	5.4	
**4Hcis**	-770.9746563	0.1684873	0.181840	0.129344	1	178.0	
**5Hcis**	-770.9757690	0.1691981	0.182962	0.129467	0	177.0	
**6Hcis**	-770.9714727	0.1681739	0.181716	0.128720	1	185.6	
**1aHtrans**	-771.6625348	0.1836874	0.197559	0.144090	0	0.0	
**3Htrans**	-771.0448138	0.1710401	0.184916	0.131193	0	0.5	-152.1
**10Htrans**	-770.9959704	0.1683435	0.182675	0.127889	0	121.7	-30.5
**(10H-H)trans**	-771.6597591	0.1829809	0.197296	0.142945	0	5.4	
**4Htrans**	-770.9737602	0.1684878	0.181829	0.129362	1	180.4	
**5Htrans**	-770.9748748	0.1691723	0.182945	0.129352	0	179.3	
**6Htrans**	-770.9706581	0.1679957	0.181597	0.128386	1	187.2	
**7Htrans**	-770.9461478	0.1675762	0.181672	0.126512	1	250.5	
**13**	-568.6961805	0.1416258	0.154021	0.102874	0	0.0	-99.7
**(13-H)**	-569.3343525	0.1545761	0.167162	0.116311	0	0.0	
**14**	-568.6520383	0.1400256	0.152059	0.102454	1	111.7	
**15**	-568.6664871	0.1408978	0.152830	0.104123	0	76.0	

**17**	-568.6727209	0.1408364	0.153044	0.102542	0	59.5	-44.1
**(17-H)**	-569.3332422	0.1556521	0.167792	0.118703	0	5.7	
**13H**	-569.2415038	0.1559037	0.168531	0.117003	0	0.0	-126.9
**(13-H)H**	-569.8687294	0.1680849	0.181078	0.129042	0	0.0	
**14H**	-569.1823114	0.1522403	0.164916	0.113665	1	145.8	19.0
**15H**	-569.1853723	0.1532281	0.166055	0.115410	0	140.3	14.0
**16H**	-569.1835730	0.1525651	0.164937	0.115095	1	143.3	15.8
**17H**	-569.1946062	0.1538261	0.166573	0.115810	0	117.7	-33.3
**(17-H)H**	-569.8591811	0.1685928	0.181272	0.131170	0	26.4	
**1Mg4H2O**	-1276.8707682	0.2755115	0.300603	0.223995	0	0.0	
**3Mg4H2O**	-1276.2121848	0.2617722	0.287155	0.209541	0	0.0	-46.9
**4Mg4H2O**	-1276.1804242	0.2592579	0.284237	0.207489	1	76.8	28.8
**5Mg4H2O**	-1276.1859157	0.2600679	0.285453	0.207917	0	64.5	17.6
**6Mg4H2O**	-1276.1838951	0.2591059	0.284242	0.207133	1	67.3	19.7
**10Mg4H2O**	-1276.2102631	0.2593527	0.285287	0.206559	0	-1.3	-34.2
**(10-H)Mg4H2O**	-1276.8742305	0.2736847	0.299725	0.221262	0	-13.9	
**1Mg3H2O**	-1200.4205935	0.2497339	0.272411	0.200735	0	0.0	
**(10-H)Mg3H2O**	-1200.4271614	0.2482019	0.271504	0.198827	0	-21.3	
**3Mg3H2O**	-1199.7641487	0.2364140	0.259168	0.186734	0	0.0	-52.0
**4Mg3H2O**	-1199.7332345	0.2336947	0.256098	0.184616	1	74.0	21.1
**5Mg3H2O**	-1199.7392633	0.2346667	0.257412	0.185289	0	60.7	8.7
**6Mg3H2O**	-1199.7368643	0.2336900	0.256167	0.184559	1	64.5	11.8
**10Mg3H2O**	-1199.7619031	0.2339310	0.257185	0.183968	0	-0.6	-30.5
**11Mg3H2O**	-1199.7186663	0.2329909	0.255694	0.183194	1	110.4	
**12Mg3H2O**	-1199.7411615	0.2332050	0.256893	0.181787	0	51.9	2.4
**1HMg4H2O**	-1277.1584907	0.2875571	0.313149	0.236238	0	0.0	
**(10-H)HMg4H2O**	-1277.1439893	0.2842331	0.313149	0.236238	0	29.3	
**3HMg4H2O**	-1276.5067137	0.2730379	0.299759	0.219393	0	0.0	-64.6
**10HMg4H2O**	-1276.4748031	0.2699830	0.297401	0.214883	0	75.8	-23.9
**1Mg2H2OOH**	-1200.0177205	0.2387685	0.259850	0.191720	0	0.0	
**(10-H)Mg2H2OOH**	-1200.0195284	0.2362982	0.258256	0.187573	0	-11.2	
**3Mg2H2OOH**	-1199.3619619	0.2254943	0.246725	0.177255	0	0.0	-53.5
**4Mg2H2OOH**	-1199.3273290	0.2220283	0.243207	0.174199	1	81.8	
**5Mg2H2OOH**	-1199.3351564	0.2227119	0.244185	0.174204	0	63.1	
**6Mg2H2OOH**	-1199.3316931	0.2216684	0.242899	0.173396	1	69.4	
**10Mg2H2OOH**	-1199.3570841	0.2219313	0.243905	0.172483	0	3.5	-38.0
**13Mg4H2O**	-1074.4169550	0.2465247	0.270485	0.195887	0	0.0	-80.2

**(13-H)Mg4H2O**	-1075.0625680	0.2594717	0.283625	0.209192	0	0.0	
**14Mg4H2O**	-1074.3813544	0.2427186	0.266947	0.191870	1	83.5	
**15Mg4H2O**	-1074.3845570	0.2440192	0.268356	0.193188	0	78.5	
**16Mg4H2O**	-1074.3719365	0.2430890	0.266979	0.192959	1	109.2	
**17Mg4H2O**	-1074.4039183	0.2455702	0.269299	0.196508	0	31.7	-25.0
**(17-H)Mg4H2O**	-1075.0718156	0.2608401	0.284169	0.212982	0	-20.7	
**3Ca4H2O**	-1753.7111031	0.2602369	0.286183	0.206041	0	0.0	-64.3
**4Ca4H2O**	-1753.6758830	0.2575668	0.255206	0.180040	1	85.5	
**5Ca4H2O**	-1753.6808380	0.2581670	0.284300	0.203467	0	74.0	10.2
**6Ca4H2O**	-1753.6786772	0.2572316	0.255204	0.179750	1	77.2	
**10Ca4H2O**	-1753.7047679	0.2573560	0.284090	0.201853	0	9.1	-35.5
**1Ca4H2O**	-1754.3625633	0.2726484	0.299118	0.217614	0	0.0	
**(10-H)Ca4H2O**	-1754.3682527	0.2718358	0.298540	0.217192	0	-17.1	
**3Mn4H2O**	-2227.0030137	0.2600383	0.286332	0.203626	0	0.0	-49.9
**4Mn4H2O**	-2226.9701740	0.2574275	0.283181	0.202340	1	79.4	
**6Mn4H2O**	-2226.9718677	0.2573593	0.283286	0.202120	1	74.7	
**10Mn4H2O**	-2226.9999322	0.2571365	0.284108	0.200529	0	0.5	-33.7
**1Mn4H2O**	-2227.6605862	0.2742922	0.299895	0.220134	0	0.0	
**(10-H)Mn4H2O**	-2227.6640818	0.2714849	0.298520	0.215008	0	-16.5	
**3Na4H2O**	-1238.5917086	0.2591431	0.285791	0.202417	0	0.0	-140.1
**4Na4H2O**	-1238.5342172	0.2580153	0.283485	0.203531	1	148.0	
**5Na4H2O**	-1238.5408460	0.2586374	0.284717	0.203454	0	132.2	
**6Na4H2O**	-1238.5385240	0.2577078	0.283363	0.203230	1	135.9	
**10Na4H2O**	-1238.5635559	0.2582064	0.284485	0.203093	0	71.5	-44.4
**1Na4H2O**	-1239.2143606	0.2736963	0.298782	0.220456	1	0.0	
**(10-H)Na4H2O**	-1239.2237869	0.2724636	0.299082	0.217000	0	-28.0	
**3Na3H2O**	-1162.1437422	0.2341230	0.258007	0.180880	0	0.0	-96.6
**4Na3H2O**	-1162.0914971	0.2315672	0.255025	0.178476	1	130.5	
**5Na3H2O**	-1162.0953932	0.2324760	0.256427	0.178749	0	122.6	
**6Na3H2O**	-1162.0925921	0.2319907	0.255319	0.180106	1	128.7	
**10Na3H2O**	-1162.1180476	0.2323099	0.256363	0.179460	0	62.7	-39.1
**1Na3H2O**	-1162.7832402	0.2477738	0.271288	0.195634	0	0.0	
**(10-H)Na3H2O**	-1162.7800836	0.2467395	0.270747	0.194717	0	5.6	
**3K4H2O**	-1676.1983170	0.2595209	0.285983	0.200659	0	0.0	-136.8
**4K4H2O**	-1676.1419209	0.2575469	0.283280	0.200938	1	142.9	
**5K4H2O**	-1676.1493400	0.2579485	0.284315	0.200826	0	124.5	
**6K4H2O**	-1676.1457916	0.2569084	0.283028	0.200060	1	131.0	
**10K4H2O**	-1676.1708131	0.2573926	0.284176	0.199871	0	66.6	-36.9

**1K4H2O**	-1676.8218751	0.2717044	0.298630	0.211703	0	0.0	
**(10-H)K4H2O**	-1676.8337519	0.2719771	0.298610	0.215024	0	-30.5	
**AdoH**	-887.6906435	0.2312361	0.248248	0.186006			
**AdoR**	-888.3634183	0.2465382	0.263125	0.202497			

Table S2. Detailed results of *ab initio* and DFT calculations. BMK/6-31+G(2df,p) optimizations.

	BMK/6-31G+G(2df,p)						
						dE	RSE
Name	Electronic energy	ZPE	cH	cG	nimag	Etot+ZPE	Etot+cH
	[Hartree]	[Hartree]	[Hartree]	[Hartree]		[KJ/mol]	[KJ/mol]
**1a**	-770.7948381	0.1731361	0.186545	0.133934	0	0.0	
**1b**	-770.7918487	0.1741983	0.187495	0.134823	0	10.6	
**3**	-770.1557182	0.1595765	0.173206	0.119650	0	0.0	-95.6
**4**	-770.1005968	0.1585211	0.171491	0.119322	1	142.0	
**5**	-770.1103175	0.1581125	0.171780	0.118159	0	115.4	
**6**	-770.1024808	0.1573918	0.170607	0.118130	1	134.0	
**7**	-770.0788944	0.1570890	0.170742	0.116602	1	195.2	
**8**	-770.1014591	0.1585074	0.173039	0.115560	0	139.7	
**9**	-770.0750589	0.1571772	0.170803	0.116981	1	205.5	
**10**	-770.1263584	0.1578370	0.171722	0.117534	0	72.5	-51.2
**(10-H)**	-770.7872829	0.1730003	0.186921	0.132688	0	19.5	
**11**	-770.1174444	0.1566697	0.170058	0.116948	1	92.9	
**12**	-770.1402944	0.1577396	0.171789	0.115958	0	35.7	
**1aHcis**	-771.3349790	0.1866726	0.200505	0.146569	0	0.0	-148.7
**3Hcis**	-770.7167386	0.1740984	0.187831	0.134452	0	0.0	-36.4
**10Hcis**	-770.6724120	0.1713404	0.185526	0.130976	0	109.1	
**(10-H)cis**	-771.3384676	0.1861132	0.200244	0.146730	0	-3.9	
**4Hcis**	-770.6532090	0.1710740	0.184321	0.132052	1	158.9	
**5Hcis**	-770.6536874	0.1715562	0.185378	0.131299	0	158.9	
**6Hcis**	-770.6493238	0.1711167	0.184551	0.131680	1	169.2	
**1aHtrans**	-771.3372400	0.1863534	0.200110	0.146957	0	0.0	-141.3
**3Htrans**	-770.7167183	0.1742781	0.187962	0.134676	0	0.5	-29.0
**10Htrans**	-770.6711990	0.1713721	0.185581	0.130988	0	112.4	
**(10-H)trans**	-771.3399587	0.1859090	0.200158	0.146085	0	-8.3	
**4Htrans**	-770.6522207	0.1715299	0.184691	0.132667	1	162.6	

**5Htrans**	-770.6526871	0.1719637	0.185581	0.132408	0	162.6	
**6Htrans**	-770.6482255	0.1706927	0.184237	0.131072	1	170.9	
**13H**	-568.9856196	0.1599458	0.172109	0.121834	0	0.0	-116.0
**(13-H)H**	-569.6150460	0.1708064	0.183540	0.132261	0	0.0	
**14H**	-568.9263053	0.1558494	0.167719	0.118975	1	145.0	
**15H**	-568.9375173	0.1558370	0.168389	0.118410	0	115.5	
**16H**	-568.9355186	0.1552693	0.167309	0.118373	1	119.3	
**17H**	-568.9412116	0.1563108	0.168882	0.118617	0	107.0	-22.4
**(17-H)H**	-569.6093995	0.1708173	0.183405	0.133584	0	14.9	
**1Mg4H2O**	-1276.4017661	0.2822638	0.306504	0.232509	0	0.0	
**3Mg4H2O**	-1275.7394525	0.2695054	0.293824	0.219736	0	0.0	-33.0
**4Mg4H2O**	-1275.7148606	0.2657566	0.290074	0.216035	1	54.7	
**5Mg4H2O**	-1275.7192015	0.2670455	0.291558	0.217101	0	46.7	
**6Mg4H2O**	-1275.7174750	0.2660831	0.290421	0.216049	1	48.7	
**10Mg4H2O**	-1275.7421977	0.2667142	0.291774	0.216051	0	-14.5	-32.4
**(10-H)Mg4H2O**	-1276.4092994	0.2807059	0.306006	0.230137	0	-23.9	
**1Mg3H2O**	-1199.9735038	0.2564810	0.277001	0.209937	0	0.0	
**(10-H)Mg3H2O**	-1199.9841622	0.2542650	0.276857	0.206062	0	-33.8	
**3Mg3H2O**	-1199.3129730	0.2424383	0.264552	0.193733	0	0.0	-37.0
**4Mg3H2O**	-1199.2898528	0.2399905	0.261606	0.192402	1	54.3	
**5Mg3H2O**	-1199.2949210	0.2402906	0.262472	0.191885	0	41.8	
**6Mg3H2O**	-1199.2927638	0.2394727	0.261410	0.191312	1	45.3	
**10Mg3H2O**	-1199.3157439	0.2397573	0.262237	0.191101	0	-14.3	-30.0
**11Mg3H2O**	-1199.2566171	0.2381532	0.260268	0.189584	1	136.7	
**12Mg3H2O**	-1199.2831089	0.2399861	0.262802	0.190158	0	72.0	36.8
**1Mg2H2OOH**	-1199.5622738	0.2439621	0.264629	0.197732	0	0.0	
**(10-H)Mg2H2OOH**	-1199.5702953	0.2423228	0.263916	0.194640	0	-25.4	
**3Mg2H2OOH**	-1198.9024901	0.2304761	0.251674	0.182137	0	0.0	-40.3
**4Mg2H2OOH**	-1198.8779847	0.2289130	0.249271	0.182200	1	60.2	
**5Mg2H2OOH**	-1198.8842648	0.2279413	0.249100	0.180207	0	41.2	
**6Mg2H2OOH**	-1198.8810298	0.2275142	0.248280	0.180322	1	48.6	
**10Mg2H2OOH**	-1198.9049254	0.2283828	0.249763	0.180335	0	-11.9	-36.8
**13Mg4H2O**	-1074.0150459	0.2530497	0.276306	0.203820	0	0	-68.8
**(13-H)Mg4H2O**	-1074.6635129	0.2653546	0.288810	0.216364	0	0.0	
**14Mg4H2O**	-1073.9879348	0.2495818	0.272525	0.201535	1	62.1	
**15Mg4H2O**	-1073.9927983	0.2513476	0.274575	0.203087	0	53.9	
**16Mg4H2O**	-1073.9770355	0.2518489	0.274032	0.204968	1	96.6	
**17Mg4H2O**	-1074.0063674	0.2515294	0.274632	0.203762	0	18.8	-26.8

**(17-H)Mg4H2O**	-1074.6772142	0.2680242	0.290470	0.221474	0	-29.0
**AdoH**	-887.9623953	0.2506013	0.266590	0.207636	0	
**AdoR**	-887.2897386	0.2356237	0.251919	0.191980	0	-11.0

Table S3. Detailed results of *ab initio* and DFT energy calculations.

	MP2/6-311++G(3df,3p)// B3LYP-6-31+G(d)			M06-2X/6-311++G(3df,3p)// B3LYP-6-31+G(d)		
Pubname	Electronic energy [Hartree]	dE Etot+ZPE [KJ/mol]	RSE Etot+cH [KJ/mol]	[Hartree]	dE Etot+ZPE [KJ/mol]	RSE Etot+cH [KJ/mol]
**1a**	-769.7679067	0.0		-771.0892815	0.0	
**1b**	-769.7657426	6.8		-771.0867597	7.8	
**3**	-769.1124231	0.0	-49.0	-770.4472803	0.0	-86.2
**4**	-769.0496581	160.0		-770.3903890	144.6	
**5**	-769.0691923	110.4		-770.4039967	110.5	
**6**	-769.0534670	149.3		-770.3919301	139.8	
**7**	-769.0337559	199.5		-770.3748222	183.2	
**8**	-769.0663607	116.6	70.3	-770.3978573	125.4	41.9
**9**	-769.0313360	206.1		-770.3707673	194.1	
**10**	-769.0892941	55.0	-24.3	-770.4167629	74.4	-43.1
**(10-H)**	-769.7633666	10.4		-771.0808282	20.7	
**11**	-769.0863712	61.3		-770.4119943	85.6	
**12**	-769.0915344	48.8		-770.4336819	29.7	
**1aHcis**	-770.3039732	6.6		-771.6292950	6.1	
**3Hcis**	-769.6751414	0.0	-117.2	-771.0083368	0.0	-139.6
**10Hcis**	-769.6359739	96.3	-22.4	-770.9633002	111.8	-30.3
**(10H-H)cis**	-770.3103347	-11.7		-771.6318025	-2.0	
**4Hcis**	-769.6067704	173.0		-770.9438884	162.7	
**5Hcis**	-769.6099247	166.6		-770.9470691	156.2	
**6Hcis**	-769.6042619	178.7		-770.9394536	173.5	
**(10H-H)trans**	-770.3103347	-11.7		-771.6318025	-2.0	
**1aHtrans**	-770.3065977	0.0		-771.6317370	0	
**3Htrans**	-769.6761920	-2.6	-113.0	-771.0083278	0.2	-133.2
**10Htrans**	-769.6356773	96.7	-16.8	-770.9623967	113.7	-23.6

**4Htrans**	-769.6065698	173.5		-770.9430853	164.8	
**5Htrans**	-769.6031981	184.2		-770.9462451	158.3	
**6Htrans**	-769.6031987	181.1		-770.9385992	175.3	
**7Htrans**	-769.5581783	298.2		-770.9060296	259.7	
**13**	-567.6529513	0.0	-42.0	-568.6469482	0.0	-81.1
**(13-H)**	-568.3108820	0.0		-569.2906999	0.0	
**14**	-567.6204216	81.2		-568.6095722	93.9	
**15**	-567.6510697	3.0		-568.6306809	40.8	
**16**	--					
**17**	-567.6412398	28.7	6.8	-568.6270738	50.1	-24.7
**(17-H)**	-568.3193667	-19.5		-569.293901	-8.4	
**13H**	-568.2037459	0.0	-68.4	-569.2011612	0.0	-110.6
**(13-H)H**	-568.8510326	0.0		-569.8330564	0.0	
**14H**	-568.1578720	110.8		-569.1484625	128.7	
**15H**	-568.1759784	65.9		-569.1581628	105.9	
**16H**	-568.1745628	67.9		-569.1567092	107.9	
**17H**	-568.1723173	77.1	-6.6	-569.1599936	102.6	-26.4
**(17-H)H**	-568.8528764	-3.5		-569.830149	9.0	
**1Mg4H2O**	-1274.7013346	0.0		-1276.8523835	0.0	
**3Mg4H2O**	-1274.0332833	0.0	-16.3	-1276.1883456	0.0	-28.6
**4Mg4H2O**	-1274.0016510	76.4		-1276.1587206	71.2	
**5Mg4H2O**	-1274.0103088	55.8		-1276.1659933	54.2	
**6Mg4H2O**	-1274.0041468	69.5		-1276.1616392	63.1	
**10Mg4H2O**	-1274.0345215	-9.6	-20.6	-1276.1869873	-2.8	-27.3
**(10-H)Mg4H2O**	-1274.7095077	-26.3		-1276.8565391	-15.7	
**1Mg3H2O**	-1198.3484795	0.0		-1200.3932758	0.0	
**(10-H)Mg3H2O**	-1198.3583853	-30.0		-1200.4005109	-23.0	
**3Mg3H2O**	-1197.6810138	0.0	-17.3	-1199.731274	0.0	-33.4
**4Mg3H2O**	-1197.6495265	75.5		-1199.7026908	67.9	
**5Mg3H2O**	-1197.6596528	51.5		-1199.7109152	48.9	
**6Mg3H2O**	-1197.6527105	67.2		-1199.705897	59.5	
**10Mg3H2O**	-1197.6824160	-10.2	-17.7	-1199.729804	-2.7	-24.0
**11Mg3H2O**	-1197.612543	170.8		-1199.675433	137.6	
**12Mg3H2O**	-1197.630574	124.0		-1199.701312	70.2	39.3
**1HMg4H2O**				-1277.1410783	0.0	
**(10-H)HMg4H2O**				-1277.1282543	24.94	
**3HMg4H2O**				-1276.4834532	0.0	-45.3
**10HMg4H2O**				-1276.4586681	57.05	-30.7

**1Mg2H2OOH**	-1197.9420448	0.0		-1199.9856596	0	
**(10-H)Mg2H2OOH**	-1197.9467226	-18.8		-1199.9860073	-7.4	
**3Mg2H2OOH**	-1197.2746164	0.0	-17.0	-1199.3245129	0	-35.3
**4Mg2H2OOH**	-1197.2384010	86.0		-1199.2902867	80.8	
**5Mg2H2OOH**	-1197.2464749	66.6		-1199.3005095	55.7	
**6Mg2H2OOH**	-1197.2467842	63.0		-1199.2936046	71.1	
**10Mg2H2OOH**	-1197.2730735	-5.3	-23.9	-1199.3181662	7.3	-37.9
**13Mg4H2O**	-1072.568111	0.0	-30.8	-1074.38742	0	-64.8
**(13-H)Mg4H2O**	-1073.230314	0.0		-1075.037349	0.0	
**14Mg4H2O**	-1072.543978	53.4		-1074.357199	69.4	
**15Mg4H2O**	-1072.558507	18.6		-1074.365812	50.2	
**16Mg4H2O**	-1072.527744	97.0		-1074.345768	100.3	
**17Mg4H2O**	-1072.558505	22.7	-30.0	-1074.36677	51.7	-25.8
**(17-H)Mg4H2O**	-1073.230314			-1075.037349		
**3Ca4H2O**	-1751.4486181	0.0	-23.1	-1753.740891	0.0	-37.6
**4Ca4H2O**	-1751.4178947	73.7		-1753.708345	78.4	
**5Ca4H2O**	-1751.4272356	50.7		-1753.715128	62.2	
**6Ca4H2O**	-1751.418792	70.4		-1753.710426	72.1	
**10Ca4H2O**	-1751.447020	-3.4	-21.3	-1753.735356	7.0	-28.2
**1Ca4H2O**	-1752.113543	0.0		-1754.400989	0.0	
**(10-H)Ca4H2O**	-1752.121754	-23.7		-1754.404585	-11.6	
**3Mn4H2O**	-2224.288002	0.0	-10.7	-2227.043245	0.0	-25.1
**4Mn4H2O**	-2224.256598	75.6		-2227.011184	77.3	
**6Mn4H2O**	-2224.256849	74.8		-2227.013201	71.8	
**10Mn4H2O**	-2224.288529	-9.0	-20.4	-2227.039516	2.2	-27.1
**1Mn4H2O**	-2224.958272	0.0		-2227.708744	0.0	
**(10-H)Mn4H2O**	-2224.963569	-21.3		-2227.709133	-8.4	
**3Na3H2O**	-1160.0472697	0.0	-62.7	-1162.081272	0.0	-81.0
**4Na3H2O**	-1159.9895805	144.8		-1162.032379	121.7	
**5Na3H2O**	-1159.9987020	123.2		-1162.042567	97.3	
**6Na3H2O**	-1159.9959596	129.1		-1162.035958	113.4	
**10Na3H2O**	-1160.0269533	48.6	-25.0	-1162.060581	49.6	-32.6
**1Na3H2O**	-1160.6974552	0.0		-1162.725197	0.0	
**(10-H)Na3H2O**	-1160.7001973	-9.9		-1162.728063	-10.2	
**3K4H2O**	-1673.9211341	0.0	-104.1	-1676.190428	0.0	-119.5
**4K4H2O**	-1673.8620048	150.1		-1676.138299	131.7	
**5K4H2O**	-1673.8746251	118.0		-1676.149664	102.9	

**6K4H2O**	-1673.8689159	128.6		-1676.144128	114.7	
**10NK4H2O**	-1673.9014484	46.1	-24.1	-1676.165247	60.5	-31.8
**1K4H2O**	-1674.5549344	0.0		-1676.819029	0.0	
**(10-H)K4H2O**	-1674.5750984	-52.2		-1676.833108	-36.2	
**AdoH**	-887.6437597		-11.5			
**AdoR**	-888.3157534					

Table S4. Important interatomic distances. Units in Ångstrom.

		C4a-C6	C4a-N5	C6-N5	C-O	C6-C9	C-Oa1	C-Oa2	N8-C7	M-CO	M-COO
**1a**	B3LYP/6-31+G(d)	2.462	1.386	1.461	1.238	1.575	1.258	1.257			
	BMK/6-31+G(2df,p)	2.446	1.384	1.452	1.223	1.575	1.243	1.243			
**1b**	B3LYP/6-31+G(d)	2.409	1.405	1.472	1.240	1.584	1.256	1.260			
	BMK/6-31+G(2df,p)	2.390	1.405	1.465	1.224	1.582	1.242	1.246			
**3**	B3LYP/6-31+G(d)	2.413	1.377	1.363	1.240	1.501	1.268	1.265			
	BMK/6-31+G(2df,p)	2.399	1.371	1.359	1.224	1.508	1.251	1.249			
**4**	B3LYP/6-31+G(d)	1.801	1.456	1.440	1.232	1.528	1.258	1.262			
	BMK/6-31+G(2df,p)	1.830	1.446	1.438	1.217	1.531	1.244	1.247			
**5**	B3LYP/6-31+G(d)	1.524	1.512	1.460	1.227	1.559	1.253	1.258			
	BMK/6-31+G(2df,p)	1.534	1.508	1.461	1.212	1.561	1.238	1.242			
**6**	B3LYP/6-31+G(d)	1.503	1.787	1.449	1.228	1.572	1.252	1.258			
	BMK/6-31+G(2df,p)	1.502	1.800	1.444	1.213	1.573	1.238	1.243			
**7**	B3LYP/6-31+G(d)	2.781	2.057	1.284	1.225	1.553	1.247	1.249			
	BMK/6-31+G(2df,p)	2.765	2.041	1.277	1.223	1.560	1.239	1.243			
**8**	B3LYP/6-31+G(d)			1.277	1.235	1.561	1.256	1.256			
	BMK/6-31+G(2df,p)			1.269	1.220	1.567	1.240	1.240			
**9**	B3LYP/6-31+G(d)	2.235	2.894	1.303	1.231	1.588	1.249	1.258			
	BMK/6-31+G(2df,p)	2.218	2.854	1.294	1.216	1.586	1.235	1.244			
**10**	B3LYP/6-31+G(d)	1.519	2.542	1.389	1.234	1.789	1.232	1.240			
	BMK/6-31+G(2df,p)	1.527	2.518	1.402	1.218	1.688	1.226	1.233			
**11**	BMK/6-31+G(2df,p)	2.417	1.392	1.327	1.228	1.554	1.243	1.238	2.125		
**12**	BMK/6-31+G(2df,p)	2.569	1.357	1.376	1.220	1.575	1.248	1.234	2.996		
**1aHcis**	B3LYP/6-31+G(d)	2.445	1.404	1.451	1.235	1.521	1.353	1.212			
	BMK/6-31+G(2df,p)	2.427	1.400	1.444	1.219	1.524	1.335	1.200			
**3Hcis**	B3LYP/6-31+G(d)	2.410	1.390	1.365	1.234	1.425	1.380	1.233			
	BMK/6-31+G(2df,p)	2.395	1.386	1.356	1.219	1.432	1.359	1.219			
**4Hcis**	B3LYP/6-31+G(d)	1.673	1.463	1.455	1.229	1.464	1.361	1.225			

	BMK/6-31+G(2df,p)	1.658	1.457	1.450	1.213	1.474	1.340	1.209			
**5Hcis**	B3LYP/6-31+G(d)	1.541	1.499	1.464	1.230	1.488	1.353	1.218			
	BMK/6-31+G(2df,p)	1.551	1.495	1.465	1.214	1.491	1.334	1.204			
**6Hcis**	B3LYP/6-31+G(d)	1.497	1.736	1.454	1.231	1.508	1.348	1.217			
	BMK/6-31+G(2df,p)	1.493	1.731	1.450	1.215	1.511	1.330	1.201			
**10Hcis**	B3LYP/6-31+G(d)	1.523	2.406	1.449	1.231	1.544	1.348	1.212			
	BMK/6-31+G(2df,p)	1.522	2.395	1.442	1.217	1.544	1.330	1.199			
**1Htrans**	B3LYP/6-31+G(d)	2.442	1.404	1.447	1.234	1.519	1.212	1.355			
	BMK/6-31+G(2df,p)	2.424	1.401	1.440	1.218	1.521	1.198	1.337			
**3Htrans**	B3LYP/6-31+G(d)	2.410	1.388	1.363	1.232	1.428	1.234	1.376			
	BMK/6-31+G(2df,p)	2.394	1.383	1.354	1.217	1.434	1.219	1.356			
**4Htrans**	B3LYP/6-31+G(d)	1.672	1.462	1.451	1.227	1.464	1.221	1.369			
	BMK/6-31+G(2df,p)	1.659	1.456	1.446	1.211	1.472	1.205	1.348			
**5Htrans**	B3LYP/6-31+G(d)	1.539	1.500	1.460	1.228	1.488	1.214	1.361			
	BMK/6-31+G(2df,p)	1.552	1.494	1.460	1.212	1.491	1.200	1.342			
**6Htrans**	B3LYP/6-31+G(d)	1.494	1.735	1.449	1.230	1.508	1.210	1.357			
	BMK/6-31+G(2df,p)	1.491	1.733	1.445	1.214	1.510	1.197	1.339			
**7Htrans**	B3LYP/6-31+G(d)	2.781	2.057	1.284	1.225	1.553	1.247	1.249			
	BMK/6-31+G(2df,p)	2.813	2.224	1.272	1.214	1.511	1.199	1.338			
**10Htrans**	B3LYP/6-31+G(d)	1.521	2.388	1.449	1.230	1.536	1.210	1.355			
	BMK/6-31+G(2df,p)	1.520	2.381	1.442	1.216	1.537	1.197	1.337			
**(10-H)trans**	B3LYP/6-31+G(d)	1.514	2.447	1.466	1.232	1.547	1.214	1.353			
	BMK/6-31+G(2df,p)	1.507	2.447	1.458	1.219	1.543	1.200	1.336			
**13Hcis**	BMK/6-31+G(2df,p)	2.500	1.373	1.361	1.214	1.452	1.356	1.215			
**(13-H)H**	BMK/6-31+G(2df,p)	2.460	1.369	1.442	1.214	1.524	1.338	1.199			
**14H**	BMK/6-31+G(2df,p)	1.511	1.877	1.433	1.214	1.529	1.331	1.199			
**15H**	BMK/6-31+G(2df,p)	1.561	1.477	1.446	1.214	1.518	1.333	1.199			
**16H**	BMK/6-31+G(2df,p)	1.522	1.488	1.458	1.214	1.518	1.332	1.200			
**17H**	BMK/6-31+G(2df,p)	1.535	2.397	1.452	1.216	1.543	1.331	1.200			
**(17-H)H**	BMK/6-31+G(2df,p)	1.535	2.491	1.461	1.216	1.539	1.328	1.203			
**13Mg4H2O**	BMK/6-31+G(2df,p)	2.436	1.409	1.399	1.239	1.468	1.299	1.241		2.039	1.969
**(13-H)Mg4H2O**	BMK/6-31+G(2df,p)	2.517	1.397	1.486	1.241	1.542	1.280	1.233		2.032	1.969
**14Mg4H2O**	BMK/6-31+G(2df,p)	1.864	1.448	1.419	1.245	1.510	1.279	1.235		2.017	1.970
**15Mg4H2O**	BMK/6-31+G(2df,p)	1.591	1.472	1.440	1.243	1.527	1.275	1.231		2.011	1.973
**16Mg4H2O**	BMK/6-31+G(2df,p)										
**17Mg4H2O**	BMK/6-31+G(2df,p)	1.526	2.404	1.443	1.238	1.596	1.270	1.218		2.030	1.995
**(17-H)Mg4H2O**	BMK/6-31+G(2df,p)	1.526	2.426	1.483	1.236	1.566	1.271	1.226			
**1Mg4H2O**	B3LYP/6-31+G(d)	2.385	1.442	1.487	1.272	1.562	1.300	1.225		2.067	2.006
	BMK/6-31+G(2df,p)	2.366	1.435	1.479	1.257	1.563	1.284	1.212		2.032	1.961
**3Mg4H2O**	BMK/6-31+G(2df,p)	2.305	1.418	1.400	1.271	1.484	1.294	1.231		2.066	1.972

**4Mg4H2O**	BMK/6-31+G(2df,p)	1.735	1.451	1.440	1.270	1.483	1.286	1.234		2.002	1.966
**5Mg4H2O**	BMK/6-31+G(2df,p)	1.528	1.526	1.466	1.272	1.512	1.276	1.229		1.991	1.984
**6Mg4H2O**	BMK/6-31+G(2df,p)	1.501	1.699	1.452	1.271	1.523	1.275	1.228		1.996	1.986
**10Mg4H2O**	BMK/6-31+G(2df,p)	1.534	2.389	1.441	1.266	1.555	1.276	1.225		2.001	1.976
**(10-H)Mg4H2O**	BMK/6-31+G(2df,p)	1.528	2.444	1.463	1.269	1.551	1.278	1.228		1.998	1.974
**11Mg3H2O**	BMK/6-31+G(2df,p)	2.404	1.400	1.409	1.291	1.496	1.304	1.241	2.185	2.025	1.979
**12Mg3H2O**	BMK/6-31+G(2df,p)	2.514	1.357	1.420	1.265	1.528	1.290	1.221	3.089	1.985	1.948

Table S5. Mulliken atomic Spin densities >0.1

		C6	C7	N8	C8a	N1	C2	N3	C4	O-C4	C4a	N5	O-COOMg
**3**		0.635			0.110		0.153						
**4**		0.414			0.250		0.242						
**5**				0.099	0.331		0.435						
**6**			0.269						0.225			0.442	
**10**		0.129							0.111			0.746	
**3Htrans**		0.510			0.114							0.190	0.105
**3Hcis**		0.454			0.214								
**4Htrans**					0.096							0.951	
**4Hcis**					0.127							0.946	
**5Htrans**					0.340		0.369						
**3Mg3H2O**	0.789										0.086		
**3Mg4H2O**	0.780			0.101						0.083	0.083		
**4Mg4H2O**	0.384		0.063	0.098		0.136		0.242	0.074				
**5Mg4H2O**	0.078		0.072	0.135		0.194		0.430	0.106	-0.088	0.118		
**6Mg4H2O**				0.139		0.132		0.346	0.098		0.346		
**10Mg4H2O**								0.101			0.959		
**11Mg4H2O**	0.302		0.267							0.149	0.218		
**12Mg4H2O**			0.149					0.119		0.384	0.240		
**3Na3H2O**	0.643										0.139		0.096
**4Na3H2O**	0.412		0.083	0.201		0.213		0.090					
**5Na3H2O**			0.108	0.252		0.366		0.178	0.089		0.088		
**6Na3H2O**				0.233		0.216		0.146			0.359		
**10Na3H2O**				0.106							0.955		
**3Ca4H2O**	0.740										0.110		0.123
**4Ca4H2O**	0.385			0.108		0.151		0.204			0.056		

**5Ca4H2O**	0.078		0.156	0.228	0.371	0.102		0.114	
**6Ca4H2O**			0.164	0.154	0.295	0.095		0.340	
**10Ca4H2O**					0.107			0.954	
**13**	0.552		0.575						
**13Mg4H2O**	0.702							0.104	
**15Mg4H2O**	0.130		1.104						
**17H**			0.112					0.975	
**3**	0.656		0.108	0.113					
**5**			0.378	0.448					
**10**								0.827	
**3Hcis**	0.483							0.217	0.162
**5Hcis**	0.122		0.384	0.321					
**6Hcis**			0.323	0.199				0.359	
**10Hcis**								0.927	
**3Htrans**	0.484							0.223	
**5Htrans**	0.135		0.394	0.318					
**10Htrans**								0.935	
**3Mg4H2O**	0.779						0.120		
**5Mg4H2O**			0.149	0.193	0.398			0.153	
**10Mg4H2O**								0.945	
**11Mg4H2O**									
**12Mg4H2O**									
**3Mg3H2O**	0.786						0.123		0.116
**5Mg3H2O**	0.106		0.136	0.171	0.437			0.152	
**10Mg3H2O**								0.946	
**3Mg2H2OOH**	0.818						0.118		
**5Mg2H2OOH**		0.100	0.259	0.327	0.209			0.122	
**10Mg2H2OOH**								0.939	
**13**	0.563		0.523						
**15**	0.158		1.008						
**17**								0.931	
**13H**	0.545		0.303					0.111	
**15H**	0.130		1.117						
**17H**			0.112					0.975	
**13Mg4H2O**	0.694						0.104	0.102	0.126
**15Mg4H2O**	0.169						0.998		
**17Mg4H2O**								0.932	

Table S6. Detailed results of *ab initio* and DFT calculations. G3B3 results.

	G3B3				
Name	Enthalp	Energy (0K)	dE Etot+ZPE [KJ/mol]	RSE Etot+cH [KJ/mol]	nimag
**1a**	-770.642442	-770.656823	0.0		0
**3**	-770.010631	-770.024792	0.0	-86.9	0
**1aHtrans**	-771.172803	-771.187029	0.0		0
**3Htrans**	-770.553838	-770.568119	0.0	-120.7	0
**10Htrans**	-770.518173	-770.532871	92.5	-20.9	0
**4Htrans**	-770.493756	-770.507487	159.2		1
**5Htrans**	-770.496355	-770.510492	151.3		0
**6Htrans**	-770.496353	-770.510491	151.3		0
**13H**	-568.847307	-568.860211	0	-100.8	0
**(13-H)H**	-569.473844	-569.474788			0
**14H**	-568.800441	-568.813332	123.1		1
**15H**	-568.807177	-568.820278	104.8		0
**16H**	-568.806786	-568.8195	106.9		1
**17H**	-568.816851	-568.817795	111.4	-20.8	0

Table S7. Calculated relative energies for the radical rearrangements of **3** complexed to different ions

	B3LYP/ 6-31+G(d)[a]	MP2[b]	M06-2X[b]
**3Ca$_{4H2O}$**	0.0	0.0	0.0
**4Ca$_{4H2O}$**‡	85.5	73.3	78.4
**5Ca$_{4H2O}$**	74.0	50.7	62.2
**6Ca$_{4H2O}$**‡	77.2	70.4	72.1
**10Ca$_{4H2O}$**	9.1	-3.4	7.0
**3Mn$_{4H2O}$**	0.0	0.0	0.0
**4Mn$_{4H2O}$**‡	79.4	75.6	77.3
**6Mn$_{4H2O}$**‡	74.7	74.8	71.8
**10Mn$_{4H2O}$**	0.5	-9.0	2.2
**3K$_{4H2O}$**	0.0	0.0	0.0
**4K$_{4H2O}$**‡	142.9	150.1	131.7
**5K$_{4H2O}$**	124.5	118.0	102.9

**6K$_{4H2O}$‡**	131.0	128.6	114.7
**10k$_{4H2O}$**	66.6	46.1	60.5
**3Na$_{3H2O}$**	0.0	0.0	0.0
**4Na$_{3H2O}$‡**	130.5	144.8	121.7
**5Na$_{3H2O}$**	122.6	123.2	97.3
**6Na$_{3H2O}$‡**	128.7	129.1	113.4
**10Na$_{3H2O}$**	62.7	48.6	49.6

Table S8. Calculated radical stabilization enthalpies the rearrangement of **3** complexed to different ions at 298.15 K.

	B3LYP/ 6-31+G(d)[a]	MP2[b]	M06-2X[b]
**3Ca$_{4H2O}$**	-64.3	-23.1	-37.6
**10Ca$_{4H2O}$**	-35.5	-21.3	-28.2
**3Mn$_{4H2O}$**	-49.9	-10.7	-25.1
**10Mn$_{4H2O}$**	-33.7	-20.4	-27.1
**3K$_{4H2O}$**	-136.8	-104.1	-119.5
**10K$_{4H2O}$**	-36.9	-24.1	-31.8
**3Na$_{3H2O}$**	-96.6	-62.7	-81.0
**10Na$_{3H2O}$**	-39.1	-25.0	-32.6

[a] All radical energies are given in kJ mol-1; absolute energies are given in the supporting information; B3LYP, ROMP2, M06-2X energies are corrected with B3LYP/6-31+G(d) enthalpy corrections (BMK values with BMK/6-31+G(2df,p) enthalpy corrections respectively).[b] basis set 6-311++G(3df,3p).

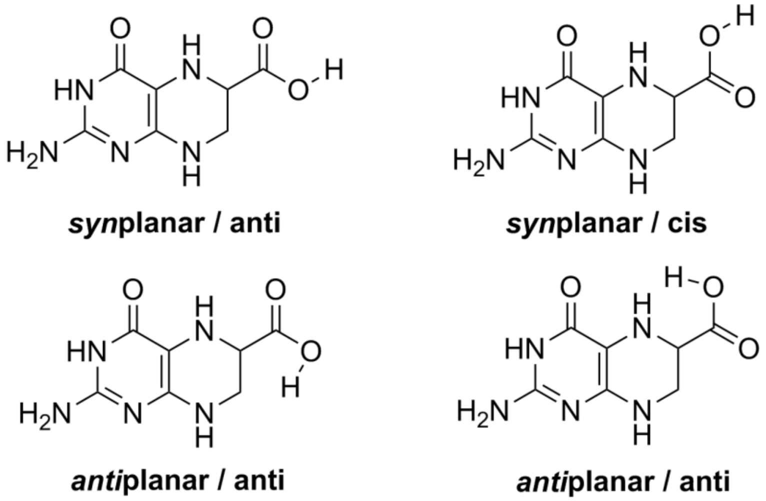

Scheme S1. Conformer nomenclature of CPH4.
